# Elucidating Ras protein as a dual therapeutic target for inflammation and cancer: a review

**DOI:** 10.1007/s12672-025-02783-x

**Published:** 2025-06-07

**Authors:** Parmar Keshri Nandan, Jayanthi Sivaraman

**Affiliations:** School of Biosciences and Technology, Vellore Institute of Technology, Vellore, India

**Keywords:** GTPases, Mutation, Cytokines, Malignancies, Apoptosis

## Abstract

Cancer, one of the most dreadful diseases of the twenty-first century develops as a result of unregulated inflammatory responses and mutations of genes. In human cancers, among various mutated genes, the Ras gene is one of the most commonly mutated oncogenes. However, when it comes to inflammation, oncogenic Ras mutants can induce tumorigenesis without tumour suppression loss driven by inflammatory responses. Ras protein is a membrane-bound protein with inbuilt GTPase activity which is activated by a variety of extracellular stimuli, converting from an inactive form to an active form. Oncogenic Ras mutants is continuously activated in cancer, thereby enhancing aberrant downstream signalling that leads to tumorigenesis. So, to highlight the significance of Ras protein in cancer and inflammation, this review discussed the signalling pathway for the activation of Ras protein, the role of Ras in immunity and inflammation, downstream regulators of Ras protein and their inhibitors epigenetic regulation of Ras protein. Moreover, we have also done a database-based search using the Database of Cancer Mutant Protein Domains (DCMP) to find out the single nucleotide polymorphism (SNP) level of the Ras gene in different types of cancer. In terms of future perspective, this review discussed emerging strategies to target Ras protein.

**Graphical Abstract:**

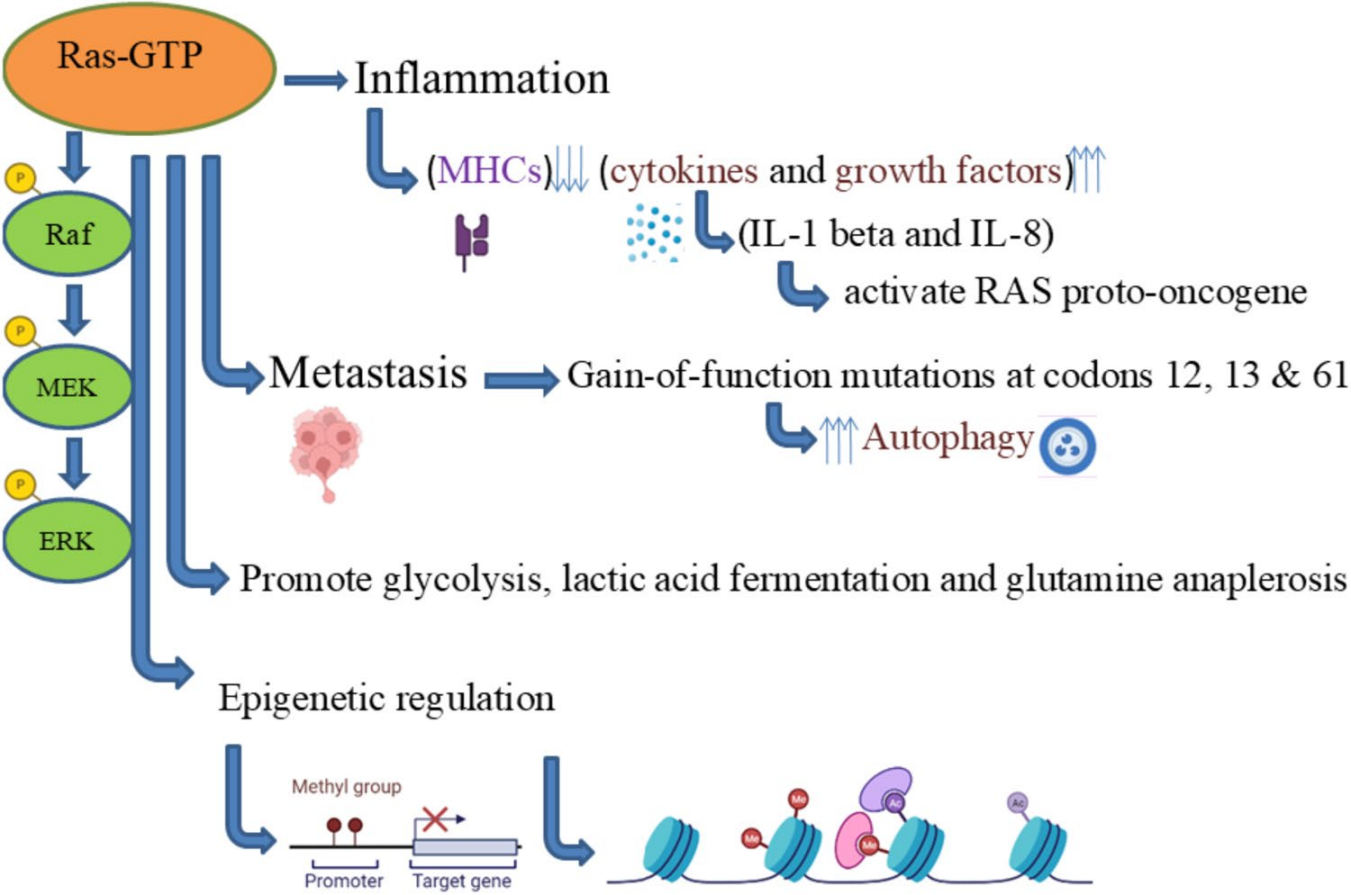

## Introduction

Ras proteins are members of the GTPase family and are thought to control cellular proliferation, migration, apoptosis, and survival. Mutant Ras proteins increase downstream signals and have a strong oncogenic function. As a result, tumor patients with mutant Ras have a poor prognosis and have shorter overall survival than those who do not have the mutation [1]. Four different Ras proteins K-Ras4A, K-Ras4B, H-Ras and N-Ras are encoded by specific Ras genes (N-Ras, K-Ras and H-Ras). Such isoforms have conserved G domains and C-terminal hypervariable regions, as well as relatively homogeneous sequences or structures (HVRs). The G domain of Ras, which consists of switch I, switch II, and a P-loop, is accountable for binding downstream effectors and transducing downstream signals, whereas the C-terminal plays an important role in Ras membrane binding [2].

The initial insight into how Ras acts like a binary switch came from the crystal structure assembly of the G-domain of HRAS in association with GppNHp, a non-hydrolysable GTP analogue. Following that, structural investigations of Ras in complex with GTPase activating protein (GAP), Guanine nucleotide exchange factors (GEF), as well as effector Ras-binding domains (RBDs), showed two switch regions and those are switch-I and switch-II, and their importance in protein–protein interactions. Between the GTP and GDP states, the two switch regions undergo conformational changes, which have been characterized by utilizing a loaded-spring mechanism [3]. Subcellular localization of Ras proteins is dictated by the isoform-specific HVRs (hypervariable region) generated by 19–20 residues on the C-terminal end, as well as the composition of local membranes, unique lipid modification and electrostatic nature of the isoform-specific HVRs [4]. Palmitoylation and prenylation of cysteine residues in the HVR promote association with membranes, which is necessary for activating downstream signalling pathways [5]. 

Ras also has various specialized tasks in normal immune cells, including downregulation of major histocompatibility complex components and overexpression of degradative enzymes, cytokines and growth factors important for haemopoietic cell growth, development, and normal function [6]. If we look into its biomarker potential then we will find that Ras mutation status could also be used as a prognostic biomarker as reported by Bahnassy et al. that K-Ras mutation status could be considered an important prognostic and predictive biomarker for colorectal cancer patients [7]. Moreover, K-Ras mutations specifically G12C mutations are prevalent in colon, pancreatic and lung cancers, so targeting K-Ras has a promising potential in ending the undruggable era of Ras protein [8]. As per the recent finding, according to a National Institutes of Health (NIH) study, mutant Ras proteins affect nuclear protein transport in addition to transmitting signals from the cell membrane. To be more precise, mutant Ras promotes the release of EZH2 from a nuclear complex, which in turn leads to the degradation of the tumor suppressor protein DLC1 and leads to uncontrolled tumor growth [9]. Conventional methods concentrate on preventing mutant Ras activation. Recent research, however, indicates that repairing the impaired self-regulation of Ras proteins may be a useful treatment approach. This approach seeks to more successfully stop the progression of cancer by restoring the protein's capacity to deactivate itself. Moreover, recent research has also demonstrated the interaction between the tumor immune microenvironment and Ras mutations. Mutant Ras affects the activation and recruitment of immune cells, which assist tumor cells to evade immune surveillance. Gaining insight into this interaction makes it possible to combine immunotherapies and Ras-targeted treatments to improve anti-tumor responses [10, 11]. 

## Ras signalling pathway

**Fig. 1:**
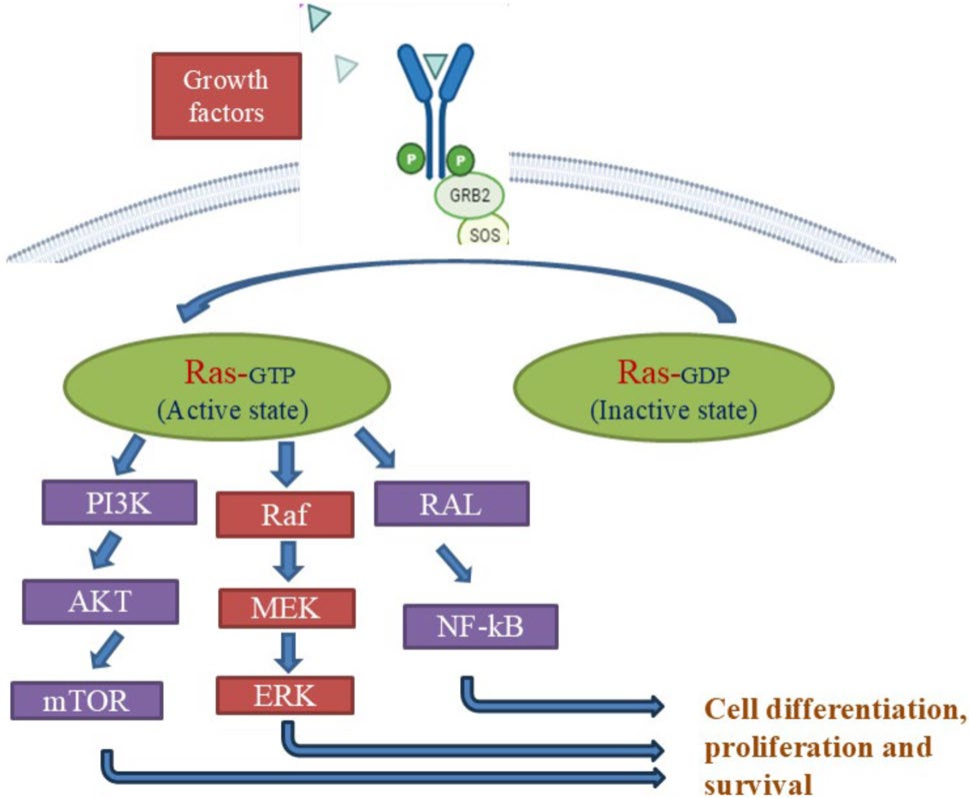
Diagrammatic representation of key steps involved in Ras signalling. Ras protein activated in presence of growth factor receptor-bound protein-2 (GRB2)—son of sevenless (SOS) in guanosine triphosphate (GTP) bound state signals in two lineages that is rat sarcoma (Ras)/rapidly accelerated fibrosarcoma (Raf)/mitogen-activated protein kinase kinase (MEK)/extracellular signal-regulated kinase (ERK) pathway and RAS/phosphatidylinositol 3-kinase (PI3K)/AKT/mammalian target of rapamycin (mTOR) pathway for cellular proliferation

The Ras proteins belong to a wide superfamily of low-molecular-weight GTP-binding proteins that may be classified into many groups based on sequence conservation [12]. Ras protein’s activation status is determined by whether they are bound to GTP (in such cases they are active and can engage downstream target enzymes) or GDP (in such cases they are inactive and cannot engage downstream target enzymes) [13]. Numerous variables control Ras activation which further has a wide range of downstream consequences. The activation and intracellular signal transduction of the Ras proteins are regulated by a variety of external stimuli, including growth factors and mitogens. Growth factor receptor-bound protein-2 (GRB2) can attract GEF to the plasma membrane, close to the receptor tyrosine kinases platelet-derived growth factor receptor (PDGFR) and epidermal growth factor receptor (EGFR) [14]. Previously it was also reported that GRB2 has massive involvement in the development and progression of different types of cancers [15]. Son of Sevenless (SOS) an exchange factor acts as the stimuli that activate Ras by switching GDP for GTP. By facilitating GTP hydrolysis, GAP contributes to the inactivation of Ras proteins under normal wild-type circumstances [16]. The next downstream targets are rapidly accelerated fibrosarcoma (Raf) and Mitogen-activated protein kinase kinase (MEK), as shown in Fig. 1. As a dual-specificity kinase, MEK phosphorylates the downstream extracellular signal-regulated serine/threonine and tyrosine sites [16] Several biological processes, including cell cycle control, apoptosis, proliferation, migration, and differentiation, are regulated by the RAS/RAF/MEK pathway [17].

Various effector proteins such as Ral guanine nucleotide dissociation stimulator (Ral-GDS), phosphatidylinositol 3-kinase (PI3K), and (Afadin) AF6 have been demonstrated to be controlled by Ras [18], as in Ras/PI3K pathway. Similarly other important Ras effectors are Ras Association Domain Family Proteins (RASSF), T-lymphoma invasion and metastasis-inducing protein 1 (Tiam1), Ras-interaction/interference proteins (RIN), Synaptic GTPase-activating protein (SynGAP), RASSF regulate apoptosis and inhibits cell proliferation [19], Tiam1 in neuroblastoma controls invasion, proliferation, and differentiation via the Tiam1/Rac1 signaling pathway [20], RIN3 a member of RIN proteins is crucial for controlling endocytosis and endocytic trafficking because it stimulates Rab5 activation [21]**,** the neural Ras GTPase-activating protein, or synaptic GTPase-activating protein (SynGAP), is greatly enriched at excitatory synapses and is expressed in the brain, where it negatively regulate Ras activity and the signaling cascades that follow [22]. 

## Ras protein and cancer

Ras family GTPase (H-, K-, and N-Ras) homologues are found throughout mammalian tissue and serve critical roles in linking extracellular inputs to numerous downstream signalling pathways. These homologues have a high degree of sequence similarity, particularly with the effector domain, which connects GTPases to downstream signalling proteins [23]. Ras genes are found to be evolutionarily conserved and they code for a monomeric G protein binding GDP or GTP. In different tumor types such as in pancreatic cancers and myeloid malignancies, mutant N-Ras, K-Ras and H-Ras occur in varying frequencies. Other members belonging to the Ras superfamily can contribute to cancer as well. Moreover, mutations could be there in downstream pathways as well, which involves PTEN, PI 3-kinase and B-Raf [24].

Among the Ras family GTPases (K-Ras, H-Ras and N-Ras), mutations in codons 61, 13 or 12 convert normally functioning genes into active oncogenes. To detect such mutations, recently rapid assays have been developed to check the role of mutated Ras genes and their relevance in the pathogenesis of human tumors. It has been reported that in a variety of tumor types Ras gene mutations can be found, nevertheless, the incidence varies significantly. Generally, 90% occurrence is reported in adenocarcinoma of the pancreas, 50% in the colon, 30% in the lung and more specifically K-Ras mutation is prevalent in about 60% of patients with non-small-cell lung cancer [25], 50% in thyroid tumors while 30% in myeloid leukaemia. Furthermore, if we look into nephroblastoma, in spite of the lack of mutations, one of the studies suggests that the Ras pathway plays an important role in the growth and proliferation of nephroblastoma [26]. In some of the tumors, a potential link may exist between the clinical features of the tumor and the existence of Ras mutation as in one of the studies presences of H-ras point mutations involves a subgroup of patients having ethmoid sinus adenocarcinomas [27]. Moreover, there is evidential support for the possible involvement of environmental agents as well in the induction of such mutations [24]. 

### Ras mutation-induced oncogenesis

**Fig. 2:**
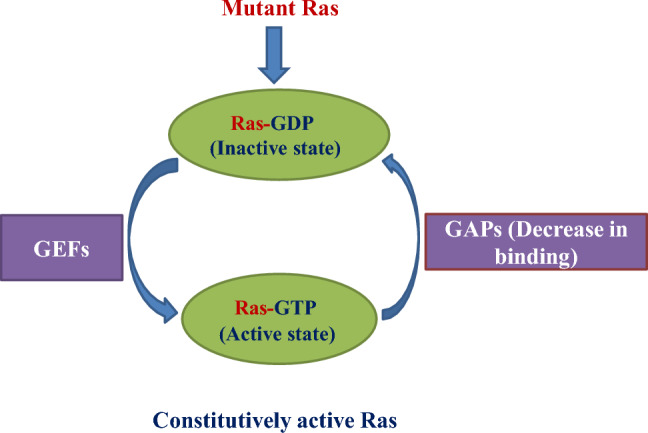
Oncogenic activation by Ras mutation. Generally, in mutant Ras, due to the rapid nucleotide exchange by guanine nucleotide exchange factors (GEFs) and decreased GTPase activating protein (GAP) binding lead to constitutive activation of Ras protein

Most gain-of-function missense mutations found in patients cluster at codons 12, 13, and 61, which enhance oncogenesis [28]. Generally, due to the rapid nucleotide exchange and decreased GAP binding, they have the effect of increasing GTP binding [29], which leads to constitutive Ras activation as shown in Fig. 2. Although in case of G12V mutant, GAP protein is insensitive to this mutant. The Ras G12V insensitivity towards GAP proteins leads to constitutive activation of Ras signaling by stopping the normal GTP hydrolysis process, and it keeps RAS in its active state [30–32]. Moreover, one more exception is there and which is with G13D mutant types, the Ras G13D mutant can promote nucleotide release without the support of guanine nucleotide exchange factors (GEFs). The G13D mutation weakens GDP binding, which leads to increased spontaneous nucleotide exchange (GDP release without GEF help). A higher proportion of Ras-GTP enhance downstream signaling. Unlike G12V, which inhibits GTP hydrolysis, G13D does not completely impair GTP hydrolysis but allows for continuous Ras activation via increased nucleotide cycling [29, 33, 34].

The potency of oncogenic Ras mutants varies and different Ras mutations are linked to different patient survival rates [35–39]. In one of the studies, CRISPR gene-editing method was used to test mutations of K-Ras by activating codons 12 and 13 concurrently in each mouse, furthermore showed that only five of the mutants led to the development of lung tumours, demonstrating *in-vivo* mutation-specific oncogenesis [40]. Additionally, it is also reported that patients' specific mutation frequency varies depending on the tissue [28]. Specific key elements interact to determine whether conditions are favourable for Ras-dependent oncogenesis to begin and proceed, which may help to explain why mutations and particular Ras isoforms are linked to particular cancer types [28]. The first is the Ras dose, which is determined by the relative activation state and expression levels [41]. Depending on the mutation present, the GTP-bound percentage of the Ras population ranges from 30 to 90% [42]. Furthermore, whether the mutant is GAP-insensitive or fast-cycling can affect how stable the active state is [29]. Ras signalling capacity can range noticeably depending on the tissue, isoform, and mutation combination, in addition to the fact that expression levels of Ras differ over 100-fold among isoforms and between tissues [43, 44]. Moreover, only a small portion of these combinations will be ideal since excessive Ras signalling encourages cell death or senescence, whereas insufficient Ras signalling prevents cancer [45–47].

It is significant to note that the limited permissive signalling capability may alter with time, promoting tumour development and drug resistance [48]. Signal specificity linked to each Ras isoform and its unique mutations is the second factor. Uncertainty exists on how differently Ras isoforms interact with effector pathways. However, to reduce dosage effects, in vivo research expressing isoforms of Ras in the same genetic locus, still indicated that isoforms of Ras cannot fully reproduce the actions of each other. This is complicated by variances in expression and dosing [49, 50]. It is believed that variable intracellular localization, which favours preferential coupling to particular effector pathways, mediates isoform-specific signalling [51–53] and by unique biochemical characteristics contributed by allosteric lobe sequence differences among each isoform [54]. Recent in vitro research revealed different binding preferences for the interactions of Ras-Raf with BRAF binding being extremely selective for KRAS and CRAF being essential for HRAS-mediated MAPK signalling [55]. Ras biology also benefits from mutational-specificity [40, 56–59] and it is currently being determined what biochemical and structural characteristics underlie mutational variations in nucleotide cycling, allosteric control, and GAP, GEF, and effector interactions [33, 42, 55, 58, 60, 61].

### Ras and cancer recurrence

The Ras protein stands as a critical player in the intricate landscape of cancer recurrence, exerting its influence through diverse cellular mechanisms. Oncogenic mutations in the Ras gene, particularly in isoforms such as KRAS, HRAS, and NRAS, have been identified in different types of human cancers, including colorectal, pancreatic, and lung cancers. These mutations contribute to the constitutive activation of Ras signalling pathways, promoting uncontrolled cell growth, survival, and metastasis. The impact of Ras extends beyond the initial stages of tumorigenesis, playing a substantial role in cancer recurrence after treatment. Studies have elucidated the linkage between Ras mutations and resistance to conventional therapies, leading to an increased likelihood of tumor recurrence and metastatic spread [62, 63].

Understanding the molecular intricacies of Ras-mediated pathways is essential for developing targeted therapies aimed at mitigating cancer recurrence. Ongoing research explores the potential of Ras-targeted treatments to impede the adaptive resistance mechanisms employed by cancer cells, offering promising avenues for preventing relapse. Clinical trials investigating Ras inhibitors and combination therapies are underway, aiming to improve outcomes and reduce the recurrence rates associated with Ras-driven cancers [64, 65]. As the field progresses, a comprehensive comprehension of Ras biology in cancer recurrence is crucial for advancing precision medicine approaches tailored to the unique molecular profiles of individual tumors.

### Ras in cancer pathogenesis and mitochondrial pathway

Oncogenic RAS increases the expression of important glycolytic enzymes, which increases the absorption of glucose and its conversion to lactate. In addition to producing ATP, this activity creates intermediates needed for biosynthetic pathways, which speeds up cell division [66]. Moreover, increased glutamine entrance into cancer cells is also made possible by RAS mutations, which upregulate glutamine transporters. Once within, glutaminase transforms glutamine into glutamate, which releases ammonia. Then, glutamate is converted to α-ketoglutarate via aminotransferases or glutamate dehydrogenase. When α-ketoglutarate enters the TCA cycle, it replenishes the intermediates needed for the synthesis of macromolecules and the production of energy [67]. Important metabolic pathways, such as the PI3K-AKT-mTOR pathway, which stimulates glycolysis and glutaminolysis, are influenced by oncogenic Ras. Through glutamine anaplerosis, this regulation guarantees a steady supply of TCA cycle intermediates, sustaining the high metabolic needs of rapidly growing cancer cells [68]. On the other hand, mitochondria primarily thought of as specific locations for energy metabolism control are unquestionably multifunctional. Mammalian mitochondrial DNA is a maternally inherited genome with high copy numbers which codes for a small number of critical proteins involved in oxidative phosphorylation. During ageing, cancer progression, and diabetes, mitochondrial DNA (mtDNA) acquires somatic mutations [69]. Recent research indicates that the Ras pathway regulates energy metabolism by controlling mitochondrial structure and function [70]. Ras proteins are engaged in a variety of signalling pathways, including cell proliferation and survival, and have been linked to oncogenic transformation due to the high prevalence of Ras mutations in cancer cells and a recent study showed Ras-mutated cancer cells had a high level of basal autophagy. This autophagy is connected to the maintenance of an effective oxidative metabolism by maintaining the pool of functioning mitochondria [71]. Another recent study found that Ras triggers autophagy by upregulating proteins such as the BH3-only proteins Noxa and Beclin 1, resulting in autophagic cell death [72] and inhibiting autophagy by knocking down Atg5 or Atg7 leading to a decrease in cell survival, which was linked to the accumulation of damaged mitochondria and the reduction in mitochondrial respiration [73].

K-Ras-dependent cellular transformation is characterized by altered mitochondrial dynamics, with oncogenic K-Ras driving mitochondrial fragmentation via extracellular signal-regulated kinase (ERK1/2) mediated activation of mitochondrial fission protein Dynamin-related protein 1 (Drp1). Drp1 inhibition or knockdown protects cells from oncogenic K-Ras-mediated transformation and slows tumor development. Furthermore, modification of the mitochondrial network as a result of oncogenic K-Ras expression has an impact on mitochondrial function, lowering membrane potential and boosting reactive oxygen species (ROS) production, thus, K-Ras-mediated mitochondrial network remodeling promotes cellular transformation by increasing tumorigenic stimuli [70].

## SNPs of Ras gene in different cancer types

**Fig. 3:**
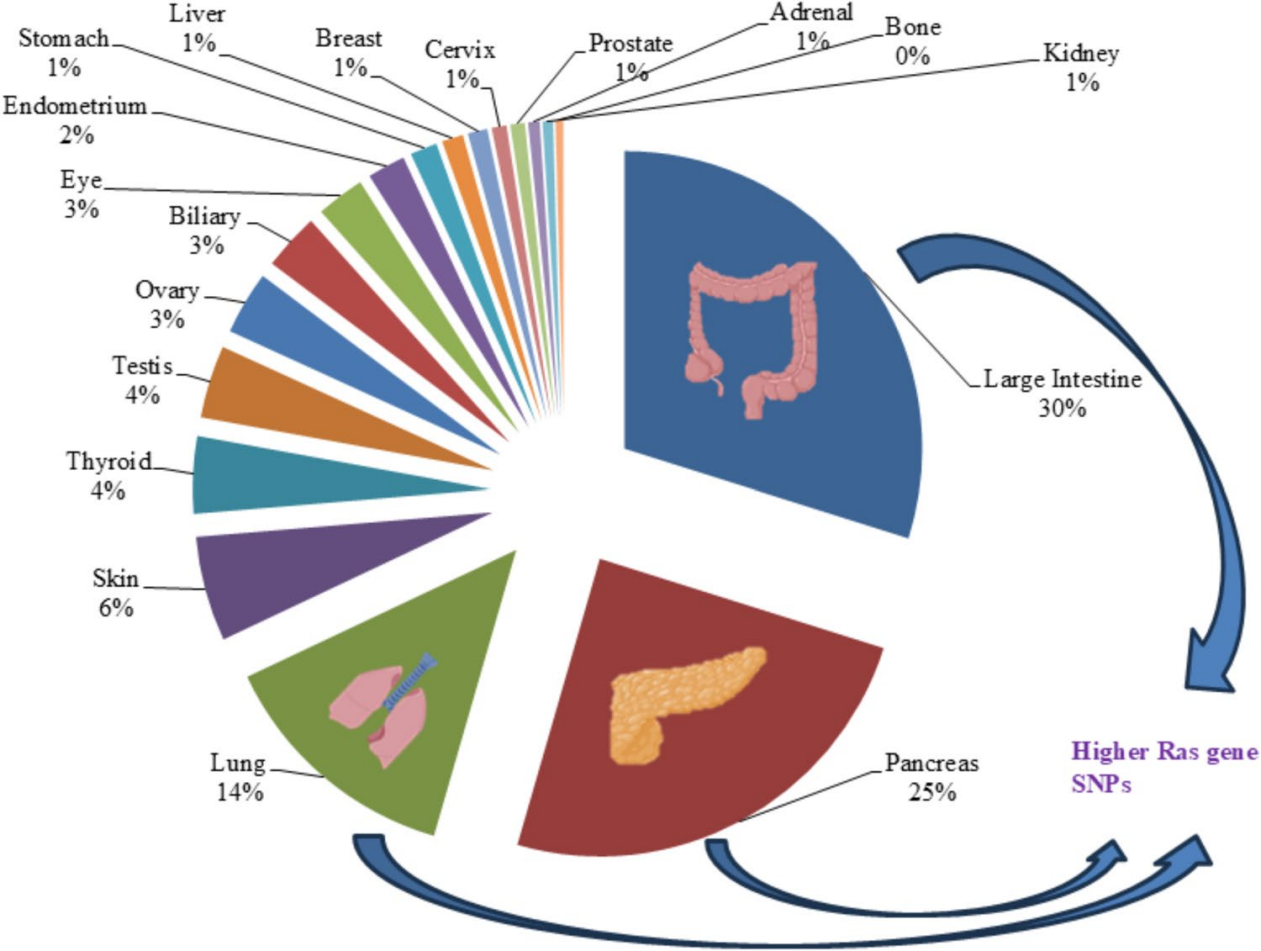
Ras gene SNPs in different cancer types

As previously mentioned, Ras gene mutation is prevalent in most cancers, so in the present study further exploration of SNP levels of Ras gene in different cancer types has been done. Database of Cancer Mutant Protein Domains [74] server was used to study the SNP level of the Ras gene in different types of cancer. SNP values for individual domain lengths of 18 different cancer types were normalized. Fetched data infer that the SNP level of Ras genes is more abundant in large intestine, pancreas and lung cancer among 18 screened cancer types as shown in Fig. 3.

## Strategies to target Ras proteins

Ras protein plays an important role in controlling the signalling pathway which is associated with cell growth and regulation as well as it also plays a key role in malignant transformation. As the Ras gene controls tumor maintenance, it is one of the suitable targets for anticancer therapy. Pharmacological inhibition is one of the prominent anti-Ras strategies which are under evaluation and designed to prevent: (1) plasma membrane association (2) upstream pathway (3) downstream signalling (4) expression of Ras [75]. It has been often observed that Ras genes are widely activated in cancer. However still, targeting specifically the mutant Ras protein has been an unsuccessful attempt. Thus, having very few targetable options, treating tumors having Ras mutations remains a difficult task to treat. Researchers are trying different Ras inhibitors such as farnesyl transferase inhibitors named Lonafarnib and other similar inhibitors but not much success has been achieved so far.

Other efforts which block activated-Ras, mainly focused on downstream pathways. For instance, those drugs which are found to inhibit Raf kinase have manifested clinical benefits while treating malignant melanoma. However, in tumors having oncogenic Ras mutants, such drugs have been clinically unsuccessful. Raf proteins bind directly to Ras and further, in normal cells, Raf proteins are major effectors of Ras action. However, for transforming activity, the extent of Ras dependency on Raf kinase remains unclear. On the other hand, for the Mitogen-activated protein kinase (MAPK) pathway, Raf kinase inhibitors (for example Raf265, AAL881, LBT613) can trigger their paradoxical activation since a question arises whether decreasing or increasing the specificity of Raf kinase inhibitors would increase therapeutic value. As far as MEK inhibitors (trametinib, binimetinib, selumetinib, and cobimetinib) are concerned, they inhibit the Ras-MAPK pathway but they also activate the PI 3-kinase and as single agents, they show little clinical benefit. Further, such activation is regulated by EGFR as well as other receptor tyrosine kinases by the relief of a negative feedback loop from ERK. Moreover, previously it is also reported that the Ras pathway activated by mutations in EGFR induces oral cancer. Mutations in the EGFR gene are common and activate various pathways such as Ras/Raf/MEK/ERK and Ras/PI3K/PTEN/AKT/mTOR, promoting transformation, differentiation, proliferation, angiogenesis and migration ultimately leading to advanced oral tumours [76].

Drug combinations which target simultaneously various points within the Ras signalling network are supposed to be important to procure substantial clinical benefit. Apart from these, other effectors such as Ral guanine nucleotide dissociation stimulator (RalGDS) and phospholipase Cε (PLCε) can also contribute to Ras signalling and consequently provide a specific source of targets. These effectors are found to interact directly with Ras proteins. RalGDS is a GEF, which stimulates the dissociation of GDP from its target Ral proteins thus allowing for the binding of GTP and subsequent activation. On the other hand, Ras signalling activation has also been associated with the breakdown of phosphoinositides through its property of binding and activating phospholipase Cϵ [77]. A rise in the active GTP-loaded K-Ras in patient samples has been linked to resistance to K-Ras G12 inhibitors, that target the inactive GDP-bound version of the protein. Dual inhibition, which targets both active and inactive K-Ras types, has been suggested as a more effective therapeutic approach to combat this. Recently explored compound MRTX1133 had strong anticancer efficacy in a number of preclinical models of colorectal and pancreatic cancer and suppressed K-Ras G12D in both its active and inactive states [78]. Furthermore, discovering small compounds that can attach to both GAP and mutant K-Ras proteins, so "gluing" them together, is a unique strategy. This method has demonstrated potential in both in vitro and in vivo settings for preventing the proliferation of cancer cells with K-Ras mutations [79]. On the other hand**,** nanoparticles-mediated gene delivery has been investigated to enhance therapeutic results in pancreatic cancer, which frequently involves Ras mutations. By directly delivering therapeutic agents to cancer cells, nanoparticles improve therapy efficacy and reduce off-target effects [80]. Moreover, by targeting particular markers of cancer cell surface, nanoparticles have made it easier to deliver chemotherapy drugs directly to tumor cells. One of the major obstacles in cancer treatment is addressed by this focused strategy, which not only increases drug deposition in cancer cells but also lowers systemic toxicity [81].

## In-silico approaches for studying Ras

The conventional approaches to achieve efficient small molecules, Ras inhibitors have not met with complete success. Thus, multi-level novel in-silico efforts for discovering potential inhibitors of allosteric sites are among the interests of researchers. In an approach, multiple steps like coupling bioinformatics analysis, ensemble docking, advanced molecular simulations as well as initial experimental testing of potential inhibitors were reported recently. In this study, the conserved allosteric connection of the nucleotide-binding switch position with distal regions has been highlighted through molecular dynamics simulation studies. Consequently, the bioinformatics approach recognized novel transient small molecule binding pockets which are close to these regions. With further cell-based analysis, it was confirmed that the downstream signalling activity of Ras was inhibited by selected binders [82].

Among H-, K-, and N-Ras proteins, K-Ras is one of the prominent types of Ras protein. It is a small GTPase which acts as a molecular switch through the recruitment of GEFs and GAPs and it alternates among the dynamic GTP-bound and inert GDP-bound forms. The amino acid present at position 12 of K-Ras is found to be the hot spot for oncogenic mutations. These mutations disturb the active fold of the protein which leads to cancer development. Mechanism-wise, it was reported that conformational changes triggered by such oncogenic mutations impair GAP-mediated GTP hydrolysis. Computational tools were used to investigate the effect of such mutations on the stability of wild-type K-Ras protein. Molecular docking followed by molecular dynamics (MD) simulation study of Ras mutations with GTP molecule was done to explore the dynamic behaviour. Finally, it was found that differential behaviours of mutant K-Ras protein complexes hindered GAP-intervened GTP hydrolysis [83].

## Downstream regulators of Ras and their inhibitors

Ras interacts with the phosphatidylinositol-3-kinase (PI3K) and Raf/MEK/Erk (MAPK) cascades, two important downstream effector signalling pathways. Specific cancer-related characteristics, such as transcriptional reprogramming, accelerated cell survival and proliferation, reduced apoptosis, and increased invasiveness of the cells are linked to these pathways [84]. Ras proteins deliver mitogenic signals to cells from the upstream growth factor receptors. Many post-translational changes make it easier for RAS proteins to bind with the plasma membrane. By incorporating an isoprenoid moiety of the farnesyl group, farnesyl transferase (FTs) makes Ras more hydrophobic and enables it for association with the plasma membrane [85]. Similarly, protein prenylation a type of post-translational modification is required for proper membrane localization and signalling. Enzymes required for the Ras prenylation process are the druggable targets for the treatment of breast cancer [86]. GTP loading activates membrane-anchored Ras, which acts as an activator to the effector kinases by luring them towards the cell membrane and further phosphorylation processes allow for their subsequent activation [84].

### Raf inhibition

Inhibition of B-Raf has been demonstrated to cause the development of Ras-dependent C-Raf/B-Raf heterodimers and to activate C-Raf, which in turn promotes carcinogenesis when oncogenic Ras is present [87]. Due to acquired or inherent drug resistance at reasonable levels, a sizable portion of patients receiving B-Raf-inhibitor and therapy developed secondary malignancies, and most frequently cutaneous squamous-cell carcinomas (cuSCC) [88]. As a result, the treatment of tumours with Ras mutations has not been successful when B-Raf inhibitors are administered alone. Instead, it has been demonstrated that combining MEK inhibitors with B-Raf inhibitors is beneficial to improve survival outcomes as well as to lower the incidence of cuSCC [89, 90]. Important B-Raf inhibitors are Vemurafenib, Dabrafenib and Encorafenib. Vemurafenib suppresses the activity of BRAF kinase by binding specifically to its ATP-binding site. It works against the cell lines having BRAF^V600E, V600D, V600R^ mutants [91]. Dabrafenib binds selectively to BRAF and stops its activity. It works against the cell lines having BRAF^V600E, V600D, V600R, V600k^ mutants [92, 93]. Encorafenib is a specific ATP-competitive inhibitor of RAF kinase. It works against the cell lines having BRAF^V600E, V600D, V600k^ mutants [94, 95].

### MEK kinase inhibition

The focus shifted to the blocking of MEK kinase because attempts to target Raf kinases have been hampered by acquired drug resistance, which restricts the efficacy of inhibitors. Prominent MEK inhibitors are Trametinib, Cobimetinib, Binimetinib and Selumetinib. Trametinib, Cobimetinib, Binimetinib and Selumetinib are important inhibitor of MEK1 and MEK2 [96, 97]. Moreover, the MEK inhibitor cobometinib has been authorized to treat histiocytic neoplasms because of its ability to effectively target mutations in the MAPK/ERK pathway [98]. In patients with N-Ras and K-Ras-mutant metastatic colorectal cancer, a phase II clinical trial examined the use of the CDK4/6 inhibitor palbociclib in combination with the MEK inhibitor binimetinib. The study revealed targetable mechanisms of resistance and identified biomarkers of response, demonstrating treatment efficacy [99]. Molecule’s selectivity is constrained since numerous inhibitors of kinase have been created that directly bind to this conserved region and compete for ATP. To increase selectivity, many allosteric inhibitors of MEK have been created. These MEK inhibitors do not bind to the MEK's ATP-binding site in a competitive manner, but rather to a specific location near to it. Allosteric MEK inhibitors, then, bind to MEK exclusively and prevent the kinase from performing its intended function by causing a conformational change which keeps the enzyme to a catalytically dormant state [100]. Low toxicity and excellent physicochemical qualities result from high selectivity. Patients with B-RafV600E/K melanoma may be treated with trametinib, cobimetinib, or binimetinib, three allosteric inhibitors of MEK [101]. The progress of combinatorial approaches has been the focus of research due to the inadequate efficiency of MEK inhibitors in the mono-agent settings. Raf inhibitors [102, 103] systemic immunotherapies [104, 105] and conventional chemotherapeutic drugs [106] have all been utilised in conjunction with MEK inhibitors in Ras-mutant malignancies to maintain a prolonged clinical benefit. A viable therapeutic strategy utilizing immunotherapy and MEK inhibitors has been suggested by the preclinical efficacy of trametinib as well as immunomodulatory antibodies in KRAS/p53-mutant lung cancer [107]. Immunocheckpoint inhibitors and MEK inhibitors together have demonstrated potential in the treatment of K-Ras-driven malignancies. This synergy is rooted in various molecular mechanisms such as immune cell infiltration, MHC Class I expression, and STING pathway activation. Inhibition of MEK can increase the infiltration of CD8⁺ T cells into K-Ras driven tumors and thus boosting anti-tumor immunity. Whereas MEK inhibition leads to increased MHC Class I expression on tumor cells which improve antigen presentation as well as cytotoxic T cells recognition. On the other hand, through the intrinsic amplification of Type I IFN signaling in tumor cells, MEK inhibition makes K-Ras driven pancreatic cancer more sensitive to STING agonism [108–110]. In one of the studies, combining trametinib with the new SOS1-KRAS interaction inhibitor BI-3406, which targets the catalytic region of SOS1, makes KRAS-driven tumours more susceptible to MEK inhibition in mice models [111]. Moreover, one recent study shows a single compound has dual inhibition activity against Raf and Mek kinases in colorectal carcinoma. In this study, the inhibitor was predicted by atomistic molecular dynamics simulations to be the top candidate for Raf and Mek protein binding. When compared to recognized inhibitors, this lead candidate demonstrated better interactions with amino acid residues and comparable binding stability with these targets. The substance successfully reduced the proliferation of HT-29 and Caco-2 cells [112].

### Erk kinase inhibition

Another target to avoid compensatory resistance mechanisms is Erk, the last kinase in the MAPK cascade. ASN007 [113], LY3214996 [114], GDC-0994 [115], and MK-8353 [116] are a few ATP-competitive Erk1/2 inhibitors that have been found, and several of them have shown considerable anti-tumor effects in cancers carrying Ras mutations. A selective ERK1/2 inhibitor called LY3214996 has shown promise in preclinical models of malignancies mediated by the ERK pathway. In cancer cells with abnormal ERK signaling, LY3214996 efficiently inhibits ERK1/2 to decrease tumor cell growth and trigger apoptosis [117]. In a phase I clinical trial involving patients with K-Ras-mutant malignancies, ulixertinib, an oral ERK inhibitor, had encouraging single-agent activity [118]. Because Erk inhibitors act as inhibitors in both normal and malignant tissues, their therapeutic index in monotherapies is still constrained. Instead, they have typically been investigated in scenarios where multiple modality therapies are used to increase their efficacy in malignancies with Ras mutations [119]. It has been demonstrated that when MEK or ERK are inhibited alone, feedback reactivation frequently results in the temporary shutdown of the MAPK pathway. A study showed that this feedback loop might be addressed by combining MEK and ERK inhibition, which would lead to longer-lasting pathway suppression and improved anticancer effectiveness in cancers with RAS mutations [120].

### Inhibition of PI3K/Akt/mTOR pathway

Tumor development and drug resistance have both been linked to abnormalities in the PI3K-Akt signalling pathway. In the axis, a subset of mutations can cause PI3K signalling dysregulation through a variety of pathways. Mutations in the subunits of PI3K (p110a and p85a), amplification of RTKs (HER2 and EGFR) deletion or mutation of the phosphatase tensin homolog (PTEN), hyperactivation of Ras or overexpression of Akt are some of these changes. Beta-catenin [121] and activated p90 ribosomal S6 kinase (RSK) [122, 123] inherent resistance makes it difficult to target Ras-induced activation of PI3K-Akt signalling alone. Previous studies show inhibitory activity of copanlisib against PI3K pathway and indicated antitumor effects in *in-vitro* and *in-vivo* experiments [124].

The second kinase member in the PI3K-mTOR axis is Akt which consists of three different isoforms that include Akt1, Akt2 and Akt3 [125]. The main function of Akt1 is to control cell proliferation and survival. Previously studies have shown that Akt1 inhibits the migration of breast cancer cells. While, Akt2 primarily promotes migration and invasion by regulating F-actin, EMT-proteins, and β-integrins. Akt2 also plays an important role in the insulin signaling pathway. In vivo studies using mouse models have revealed the opposing roles of Akt1 and Akt2 in cell invasion and migration. Whereas, Akt3 has been linked to brain development and is mostly expressed in the brain. Akt3's function in neural growth is demonstrated by the smaller brain size in mice lacking Akt3 expression. Furthermore, Akt3 has been linked to a number of malignancies, such as prostate and breast cancers, indicating a role in tumor progression [126, 127]. Different Akt isoforms have different targets for inhibition based on their unique functions in cellular processes and disease progression. As Akt1, Akt2, and Akt3 have different and occasionally conflicting activities, selective inhibition techniques need to be carefully designed to optimize therapeutic advantages and minimize undesirable side effects. Akt1 inhibitors may be helpful in malignancies when Akt1 drives survival, such as in lung cancers. Since Akt2 overexpression is associated with worse outcomes in metastatic tumors, selective inhibition of Akt2 may be helpful, particularly in breast and ovarian cancers. Akt3 inhibition may slow the growth of brain tumors. However, brain-penetrant inhibitors with high specificity are desirable because systemic Akt3 inhibition may result in neurodevelopmental problems or cognitive impairments [128–130].

**Fig. 4:**
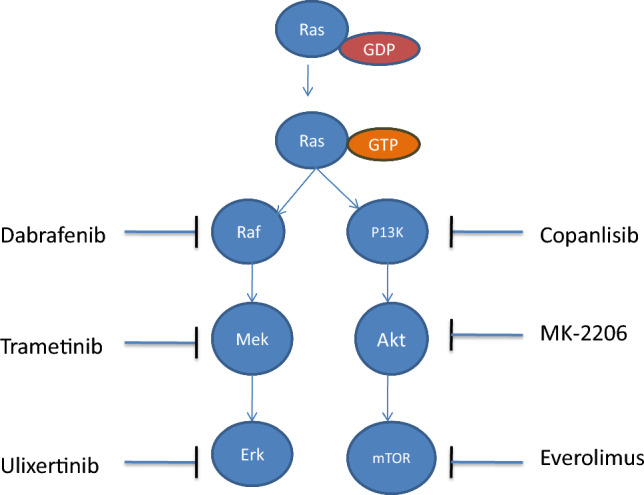
Downstream regulators of Ras protein with their prominent inhibitors. Downstream regulators of Ras protein are rapidly accelerated fibrosarcoma (RAF), mitogen-activated protein kinase kinase (MEK), extracellular signal-regulated kinase (ERK), phosphatidylinositol 3-kinase (PI3K), Akt and mammalian target of rapamycin (mTOR) and these are inhibited by Dabrafenib, Trametinib, Ulixertinib, Copanlisib, MK-2206, Everolimus respectively

All three Akt isoforms that is Akt1, Akt2, and Akt3 are targeted by the ATP-competitive inhibitor capivasertib. Capivasertib has shown anticancer effectiveness in a variety of cancer types through the inhibition of Akt phosphorylation, which results in increased apoptosis and decreased cell proliferation [131]. Another allosteric Akt inhibitor that blocks Akt activation and downstream signaling is miransertib, which targets the PH domain. By inhibiting cell division and triggering apoptosis, it has shown promise in the treatment of a number of malignancies [132]. In one of the studies, the proliferation of cancer cells with K-Ras mutations was inhibited by the combination of an allosteric inhibitor of Akt (MK-2206) and several RTK inhibitors (jnj38877605, Lapatinib, OSI-906) [133]. Moreover, clinical trials have also been conducted with inhibitors that target mTOR, one of the effectors of RAS in downstream signalling; nevertheless, the bulk of these single-agent treatments failed to produce obvious clinical advantages [134, 135]. Recently one of the studies reported that osteosarcoma malignancy is suppressed by Chitosan oligosaccharide through PI3K/Akt/mTOR pathway by the inhibition of cell migration-inducing protein (CEMIP) [136]. In patients of RAS/Raf-mutant solid tumours, a phase one trial which is based on the combination of a dual Raf/MEK inhibitor, as well as everolimus (mTOR inhibitor) (NCT02407509), is now being conducted [119]. Figure 4 and Table 1 shows prominent inhibitors of downstream regulators of Ras protein, their mechanism of action, clinical trial status and potential therapeutic uses.

**Table 1:** Prominent inhibitors of downstream regulators of Ras pathway

Inhibitors	Mechanism of action	Clinical trial status	Potential therapeutic uses
Raf inhibitor (Dabrafenib)	It selectively inhibits mutant BRAF V600E, and block downstream MEK/ERK signaling	Approved for clinical use	Treatment of melanoma, NSCLC, and anaplastic thyroid cancer with BRAF V600E mutations
MEK inhibitor (Trametinib)	Inhibits MEK1/2, preventing ERK1/2 activation and blocking downstream signaling	Approved for clinical use	Used in combination with BRAF inhibitors for melanoma, NSCLC, and thyroid cancer with BRAF V600 mutations
ERK inhibitor (Ulixertinib)	Selectively inhibits ERK1/2, blocking MAPK pathway activation	Undergoing clinical trials	Investigational use in cancers with MAPK pathway mutations, including melanoma and colorectal cancer
PI3K inhibitor (Copanlisib)	Inhibits PI3K-alpha and PI3K-delta isoforms, reducing AKT signaling and tumor growth	Approved for clinical use	Treatment of relapsed follicular lymphoma
AKT inhibitor (MK-2206)	Allosterically inhibits AKT, reducing phosphorylation and activity of downstream targets	Undergoing clinical trials	Investigational use in solid tumors, breast cancer, and hematologic malignancies
mTOR inhibitor (Everolimus)	Inhibits mTORC1, reducing cell proliferation, angiogenesis, and metabolism	Approved for clinical use	Treatment of advanced renal cell carcinoma, breast cancer, pancreatic neuroendocrine tumors

## Role of Ras in immunity and inflammation

Ras signalling has been demonstrated to be the essential component of immune cell signalling, regulating proliferation, maturation and activation of many cell types. Antigen receptor on T cells in animals carrying a dominant-negative type of H-Ras in the T lineage cells, TCR (T- Cell Receptor) stimulation promotes fast activation of Ras, and positive selection of immature CD4/CD8 double-positive T cells to mature single positive cells is repressed [137]. When it comes to RAS biology, it is still poorly understood, despite being one of the most commonly mutated signalling pathways in cancer patients, dysregulation in RAS signalling has a direct impact on not only the development of numerous pathological conditions in infected cells but also on host physiological processes including the immune response to viral infection and inflammation [138].

The biology behind the development of inflammation is closely related to macrophages. Macrophages are an important element of the mononuclear phagocyte system, which is formed of bone marrow cells such as blood monocytes and tissue macrophages, these monocytes move from the blood to various tissues and transform into macrophages which play a vital role in the development, resolution and maintenance of inflammation [139]. To maintain the body’s homeostasis, macrophage phagocytic function is generally associated with the engulfment of dying cells, foreign particulates and pathogens. Ras-related binding (Rab) GTPases are the most numerous branches of the Ras-related small GTPase superfamily, and they can precisely regulate receptor intracellular trafficking, including the movement of newly formed receptors from the endoplasmic reticulum (ER) to the cell surface, endocytosis of receptor-ligand complexes from the cell surface, and translocation of the complexes to the endosomes. Rab43, a member of the Rab family, is thought to be involved in the transport of G protein-coupled receptors (GPCRs). A study has proven, that suppressing anterograde transport of CD91 (cluster of differentiation 91) and HMGB1 (High-mobility group box 1) regulated by Rab43, which impairs macrophage-mediated efferocytosis delays inflammation resolution, suggesting that restoring Rab43 levels is a promising approach for attenuating inflammation in humans [140].

Two inflammatory mediators which closely related to the activation of Ras proto-oncogene are IL-1 beta and IL-8. IL-1beta is a pro-inflammatory cytokine linked to pain, inflammation, and autoimmune diseases. Elevated IL-1 beta levels have been linked to tumour initiation, invasiveness, and progression in a variety of cancers. Indeed, IL-1 beta is abundant at tumor sites, including lung tumors; high levels of IL-1beta were discovered in the serum and tissues of lung cancer patients and were linked to a poor prognosis. In the case of inflammatory-mediated cancer, K-ras-mutant lung adenocarcinoma (KM-LUAD) has a poor prognosis and is closely linked to tumor-promoting inflammation. In combination with currently available treatments for KM-LUAD (K-ras-mutant lung adenocarcinoma), IL-1beta blockade may be a preventive approach for high-risk individuals and an alternative therapeutic approach [141]. Similarly, there are other important cytokines which are involved in Ras-associated inflammation and cancer as shown in Table 2.

**Table 2:** Important cytokines involved in Ras-associated inflammation and cancer

Cytokines	Description	References
Tumor Necrosis Factor-alpha (TNF-α)	Up-regulation of E-selectin and vascular adhesion molecule-1 in endothelial cells caused by TNFα is blocked by inhibiting the expression of c-raf and Ha-ras	[158]
Interleukin-1 (IL-1 α)	Development of pancreatic ductal adenocarcinoma requires K-Ras G12D-induced IKK2/b/NF-kB activation through IL-1a and p62 feedforward loops	[159]
Interleukin-8 (IL-8)	In Ras signalling, the inflammatory mediator interleukin-8 (CXCL-8/IL-8) is a transcriptional target. It has also been shown that Ras-dependent CXCL-8 secretion is necessary for the initiation of tumor-associated neovascularization and inflammation	[160]
Interleukin-6 (IL-6) and Monocyte Chemoattractant Protein-1 (MCP-1)	To look into how Ras-induced cellular senescence contributes to atherogenesis, proinflammatory cytokines and chemokines were examined in human VSMCs. The expression levels of proinflammatory cytokines and chemokines, including IL-1β, IL-1α, IL-8, IL-6, and monocyte chemoattractant protein (MCP-1), were significantly increased upon the introduction of H-rasV12	[161]

Inflammation and wound healing during stress and injury are intricate biological responses involving complex interactions among different cell types that control the expression of different biological mediators. These secreted cytokines promote cell activation and proliferation, along with chemokines that induce chemotaxis and cell migration. Similar signalling events are seen in cancer cells also. Sparmann and Bar-Sagi reported that activating Ras proto-oncogenes in cancer cells causes overexpression of the inflammatory cytokine interleukin-8 (IL-8) which is a powerful proinflammatory chemokine that can cause neutrophils to rapidly migrate to infection and inflammation sites, which promotes tumour inflammation, vasculogenesis, and, finally, tumour development [142]. Moreover, research has shown that the MAPK pathway can be used by Ras proteins, such as H-Ras, to stimulate the release of interleukin-6 (IL-6) and interleukin-8 (IL-8). It has been demonstrated that inhibiting ERK, a downstream element of the MAPK pathway, lowers the secretion of IL-6 and IL-8, indicating that this route mediates Ras-induced cytokine production. Additionally, previous studies shows that K-Ras mutations can raise the expression of IL-6 and IL-8 in vivo. These results imply that oncogenic Ras signaling, including that driven by H-Ras or other subtypes can trigger the in vivo release of IL-6 and IL-8, which aids in tumor growth and immune regulation [143–145].

Chronic inflammation is caused by many factors such as bacterial, viral, and parasitic infections, chemical irritants, and indigestible particles which directly activate leukocytes, like neutrophils, monocytes, macrophages, and eosinophils thus producing soluble factors that are thought to play a role in the development of inflammation-related cancer. The longer the inflammation lasts, the greater the risk of carcinogenesis [146]. A central paradigm in carcinogenesis is the acquisition of an activating RAS signalling pathway mutation and loss of tumour suppression, which is commonly caused by p53 mutation or deletion. Inflammation allows mutant RAS to avoid the requirement for tumour suppression loss to drive tumorigenesis [147]. Pancreatic ductal adenocarcinoma (PDAC), the most Ras-addicted cancer [148] is a deadly disease with an extremely poor prognosis. Inflammatory processes have emerged as key players in the development and progression of pancreatic cancer [149]. It is unclear whether systemic inflammation is solely caused by the existence of invasive PDAC or if it develops in conjunction with the inflammatory pancreatic microenvironment of precancerous intraepithelial neoplasia (PanIN). Indeed, recruited M1 macrophages appear to enhance not only reversible ADM (acinar-to-ductal metaplasia) but also KRAS activation associated with pre-neoplastic lesions of PanINs, as well as additional IL13-dependent steps toward PDAC development [150].

The NF-B pathway is the major regulator of inflammation and is closely associated with cancer as a pro-survival and oncogenic pathway. The inhibition of NF-B in myeloid cells or tumour cells causes tumour regression. Both the Baldwin and Jacks laboratories demonstrated that the NF-B pathway collaborates with oncogenic Ras to enhance tumorigenesis in non-small cell lung cancers (NSCLC) [151]. Inflammation maximizes the likelihood of the constant occurrence of processes leading to fibrosis. Indeed, an inflammatory infiltrate is a characteristic of the initial phases of fibrosis. Inflammatory cells can transform fibroblast metabolism which may profoundly affect ECM production and collagen synthesis by direct cell–cell interactions and/or by secreting soluble products such as TGF-β, connective tissue growth factor (CTGF), interleukin (IL)-6, altered Ras signalling. One of the best examples of this phenomenon is systemic sclerosis (SSc) commonly known as connective tissue disease with a high morbidity and mortality rate, characterized by severe fibrosis affecting various organs such as the lungs, dermis and heart [152].

The RalB GTPase pathway, a crucial effector of oncogenic Ras signaling, is involved in the Ras-mediated activation of TANK-binding kinase 1 (TBK1). RalB is activated in this pathway as a result of active Ras's interaction with the Ral guanine nucleotide dissociation stimulator (RalGDS). RalB recruits and activates TBK1 through its interaction with Sec5, a part of the exocyst complex. Once activated, TBK1 stimulates the generation of cytokines, autophagy regulation, and NF-κB signaling, all of which support the survival of cancer cells and immune evasion. Since TBK1 is a major downstream modulator of oncogenic Ras and a possible therapeutic target, this signaling pathway is especially important in KRAS-driven malignancies like pancreatic cancer [153, 154].

Tendinopathy is a chronic disorder that affects a large population and has significant socioeconomic consequences worldwide. Tendinopathy was found to be more common in diabetic patients with greater chronic inflammation, the high glucose environment in diabetic patients may cause chronic inflammation in the tendon and, eventually, the development of tendinopathy [155]. High glucose levels increase inflammation by creating a pro-inflammatory environment via the production of some pro-inflammatory mediators (cytokines, chemokines, and leukotrienes), and by influencing the recruitment of immune cells, leukocytes in the inflamed region which eventually stimulates epidermal growth factor (EGF), that binds to the EGF receptor (EGFR) and promotes cancer cell proliferation via the PI3K/Akt/mTOR and Ras/Raf/MEK pathways [156]. Moreover, in another work focusing on the downregulation of inflammatory responses using the Ras pathway, it was found that Alpha-lipoic acid reduces atherosclerotic lesions and inhibits vascular smooth muscle cell proliferation by targeting the Ras/MEK/ERK signalling pathway [157].

## Ras and epigenetic silencing

Mutations can lead to cancerous developments in the body. In consideration of the role performed by epigenetics in Ras proteins, it is clear that this can be the cause of certain cancers. External stimuli and cellular behaviors to niche can influence the Ras gene family thereby altering cell growth and death patterns leading to cancerous cell development. Hence, epigenetic changes increase the chances of cancer.

Epigenetic mechanisms act as regulators for gene expression and are usually altered in human cancer development. Epigenetic mechanisms include DNA methylation, histone modifications as well as small and long non-coding RNAs associated with the regulation of pro-oncogenic pathways in cancer. Ras activation is regulated by epigenetic signature including hypomethylation of CpG islands [162] increased histone deacetylase activity in the Ras promoter, and translational repression by miRNAs.

Of the epigenetic changes, when overall DNA methylation increases, there results in decreased expression of a set of genes thereby increasing cancer risks. Although the cancerous cells will show signs of heightened DNA methylation, the overall amount is much lesser than the DNA methylation of regular cells. Epigenetics can help one to study the type of cancer existing in a patient or even help in early detection. With the context of a specific and systemic pattern of epigenetic variations, however, this alone is not a perfect choice to diagnose cancer entirely, and thus further screening tests will be required. It is known that a cancerous cell develops through changes that are influenced either by the repression of tumor suppressors or the activation of oncogenes [163].

In a study revolving epigenetic silencing of Ca2 + regulated RASAL (Ras GTPase activating protein or Ras GAP), a new mechanism of Ras activation was observed that led to cancer. RASAL protein is a Ca2 + Ras GAP that is known to be involved in the decoding of CA2 + oscillations. In this epigenetic silencing, the RASAL protein is silenced by CpG methylation in multiple tumors. As Ras GAP is involved in switching off Ras signalling, they are identified as tumor suppressor genes. Hence, the epigenetic silencing of this results in atypical Ras activation in cancers. Another observation was that tumor cell growth inhibition was observed by Ras inactivation when catalytically active RASAL is expressed ectopically [164].

As mentioned earlier, Ras-mediated epigenetic silencing of many different and unrelated genes takes place through a complex and specific pathway that involves various elements to maintain the completely transformed phenotype. This was proven in observations from a genome-wide RNA interference (RNAi) screening in K-Ras- (a member of the Ras family) -transformed NIH 3T3 cells. This screening identified about 28 genes that were needed for Ras-mediated epigenetic silencing of the Fas gene (pro-apoptotic gene). In 9 of the Ras epigenetic silencing effectors (RESEs), they were found to be directly linked with certain regions of the Fas promoter in K-Ras transformed NIH 3T3 cells. However, this was absent in untransformed cells. Even the presence of one RNAi-mediated knockout of any of the 28 RESEs led to DNMT1 (DNA methyltransferase) recruitment failure and subsequently loss of Fas promoter hypermethylation and thus Fas expression repression is revoked. Another analysis involving 5 other epigenetically repressed genes showed that the aforementioned statement was true [165]. Furthermore, list of prominent epigenetic markers involved in regulating Ras signaling in various cancers has been shown in Table 3.

**Table 3:** Prominent epigenetic markers involved in regulating Ras signaling in various cancers

Epigenetic alteration	Epigenetic marker	Mechanism	Effect on Ras pathway	Cancer types	References
DNA hypermethylation	DUSP6 (hypermethylation), RASSF1A (hypermethylation)	Block Ras inhibitors	Sustained Ras activation leading to uncontrolled cell growth	Pancreatic, lung, liver, colorectal	[166, 167]
Histone modification (H3K27me3 by EZH2)	H3K27me3 (EZH2 overexpression)	Represses tumor suppressors	Enhances RAS-MAPK signaling	NSCLC, breast, prostate	[166, 168]
miRNA dysregulation	miR-137 (downregulated), miR-143/145 (downregulated)	Loss of miRNA-mediated KRAS inhibition	Rise in K-Ras expression leading to tumor progression	Colorectal, pancreatic, lung	[166, 169]
Chromatin remodeling defects	SMARCA4 (mutation/loss), ARID1A (mutation/loss)	Alters chromatin accessibility	Upregulates oncogenic RAS effectors	Gastric, ovarian, lung, liver	[166, 170]

### DNA methylation

Ras activation is effectively regulated by specific epigenetic signatures including hypomethylation of CpG islands within Ras promoters [162], increased histone deacetylase activity in the Ras promoter, and/or translational repression by miRNAs. In addition, activation of the Ras gene also regulates locus-specific methylation and leads to transcriptional activation of many genes. Oncogenic activation of Ras controls DNA methylation by inducing DNMT1 expression at the transcriptional level by activating cJUN [171]. Along with an increase in DNMT expression, ten-eleven translocation enzymes (TET1) are also transcriptionally downregulated via the Ras/ERK pathway [172]. The excess of DNMT and the reduced TET1 levels would target certain genes for hypermethylation. It has been reported that promoters associated with aberrant CpG methylation of the tumor suppressors and apoptotic genes by DNA methyltransferases are regulated by Ras GTPase signalling. Several lines of evidence suggest that RASSF1 (Ras Association Domain Family Protein 1), a tumor suppressor gene that controls the Ras pathway is one of the most commonly reported hypermethylated genes in more aggressive cancer types and a driving force behind the progression of drug resistance [173]. Moreover, Ras along with tumor suppressor protein phosphatase 2A regulates DNA methylation, histone methylation, and histone deacetylation via dephosphorylation of these epigenetic complexes involved in aberrant tumor progression [174]. Yet another mechanism dysregulates epigenetic signature and increases the rate of mutation of Ras. Repair methyltransferase MGMT (O6-methylguanine-DNA methyltransferase) promoter hypermethylation and transcriptional silencing promote an increased rate of mutation of K-Ras.

One of the studies discusses the vital role of an important hydroxylation enzyme named Ten-eleven translocation enzymes in affecting the K-Ras functions. In Ras-dependent tumorigenesis, the silencing of the tumor suppressor gene triggered by hypermethylation is a central epigenetic modification. DNA methylation can be depressed by TET enzymes through hydroxylation of 5-methylcytosine (5mC) bases to 5-hydroxymethylcytosine (5hmC). It was reported that for cellular transformation and K-Ras-induced DNA hypermethylation suppression of TET1 is essential. In this study upon inhibition of TET1 expression, ERK-signalling mediates transformation through oncogenic K-Ras. Mechanism-wise, chromatin occupancy at TSG promoters of TET1 is reduced and consequently, it decreases 5hmC levels. This increases the levels of 5mC and finally in 5mC-dependent transcriptional silencing. Moreover, ERK pathway inhibition restores TET1 expression, and reintroduction of ectopic TET1 in K-Ras-transformed cells inhibits colony formation and reactivates TSGs. Similarly, it was also found that TET1 expression is increased and colony-forming ability is diminished by K-Ras knockdown [172].

### Acetylation

Like methylation, another frequent Post translation modification which is associated with contributes in the development of disease is acetylation, where an acetyl group from the metabolite acetyl-coenzyme A is transferred to either the amino group at the N-terminus of a protein or the -amino group of lysine residues [175]. Although Ala at Lys104 (which is non-acetylatable) showed no influence on cell proliferation compared to cells expressing unsubstituted K-Ras, K-Ras was previously believed to be acetylated at this position (G12V) [176]. However, in NIH3T3 cells, K-Ras (G12V) with the K104Q mutation (which mimics acetylation) showed decreased GEF-induced nucleotide exchange and decreased transforming activity [177]. Other than this, acetylation was reported in two more places Lys104 and Lys147 [178]. Thus, it looks like location-specific and multilateral acetylation-like epigenetic events determine the Ras signalling modulations.

On the other hand, the genetic-code extension method used to incorporate acetylated lysine into K-Ras showed that changing this residue did not affect the modified protein's SOS-mediated nucleotide exchange [178]. According to these findings and other research on Lys 104 mutations, glutamine is a low-key partner for acetylation at this location, given the significance of Lys 104 in preserving the structural integrity of helices 2 and 3, the hypothesis that acetylation controls Ras activities is still a possibility [179]. According to recent research, K-Ras is acetylated on the N-terminal Thr followed by the removal of the initial Met. Such alteration stabilises the N-terminus and switch regions by interacting with a central beta-sheet. K-Ras loose this modification whereas the initial Met take on an open, inactive, nucleotide-free conformation [180].

## Conclusion

On molecular details, we have learned enough about the Ras gene just over 30 years after it was discovered, that it was an active oncogene in human tumors. Ras proteins are small molecules that regulate a wide range of biological activities. They are found at the intersection of membrane receptors and multiple signalling pathways. Ras works in part by attracting proteins to the plasma membrane, where they become activated. The incorporation of Ras isoforms into immunological signalling complexes can be governed by their affiliation with certain membrane subdomains or assembly factors, as well as their access to specific activators GEFs or deactivators GAPs, this might alter how well each can activate specific effector pathways. The Ras signalling pathways, like many other signalling pathways under investigation by the pharmaceutical industry, are essential for the survival of both normal and tumor cells, even though tumor cells are more dependent on them. New techniques of creating chemicals based on structural considerations and a better knowledge of Ras processing and membrane localization are being pursued in an attempt to directly assault Ras proteins. These initiatives are still in the early stages of medication development. Targeting downstream pathways, on the other hand, is becoming a key focus of clinical research since a large pipeline of therapeutic candidates targeting proteins in the MAPK and PI3'-kinase pathways is being evaluated in clinical trials. Understanding the structure, function, and changes in the Ras protein, as well as employing various approaches has aided in drug discovery and should continue to lead to novel possible therapies.

## Future perspective of targeting Ras protein in inflammation and cancer therapeutics

The prospects of targeting Ras proteins in cancer therapeutics and inflammation are promising, marked by ongoing research and innovative developments. Emerging therapeutic strategies focus on addressing Ras mutations, particularly in K-Ras, as a central theme in cancer drug discovery. Advances in precision medicine and the exploration of novel Ras inhibitors hold the potential for more effective and personalized treatment options. Additionally, as Ras proteins are implicated in inflammatory processes that contribute to cancer progression, targeting Ras-related inflammation presents a dual opportunity for therapeutic intervention. The intersection of cancer and inflammation indicates the significance of understanding Ras biology in both contexts. Furthermore, recently researchers have created algorithms to create novel K-Ras inhibitors using quantum computing. This strategy has produced promising compounds that interact with oncogenic K-Ras mutants in an efficient manner, providing a possible means of overcoming resistance. This breakthrough shows the future potential of quantum computing to create experimentally validated hits [181]. Innovative methods are being explored to restore the impaired function of mutant Ras proteins. Researchers are looking for ways to restore the protein's capacity to appropriately regulate cell growth rather than only blocking RAS function. By addressing the fundamental flaw causing cancer to spread, this approach may result in more effective therapies. On the other hand, recent studies demonstrate the intricate connection between the tumor immune microenvironment and Ras signaling. In addition to aiding in tumor immune evasion, mutant Ras proteins can affect immune cell activation and recruitment. Comprehending this interaction creates opportunities to combine immunotherapies and Ras-targeted treatments to improve anti-tumor responses [11]. Moreover, according to recent research, mutant Ras contributes to genomic instability by causing stress on DNA replication. A possible therapeutic approach is to target the processes that cause this stress, as this could make RAS-mutant cancer cells more sensitive to particular therapies [182]. Ongoing clinical trials, such as those investigating Ras inhibitors, continue to pave the way for transformative approaches in cancer therapy, offering hope for improved outcomes and novel anti-inflammatory strategies. The evolving view of Ras-targeted therapeutics holds promise for shaping the future of cancer treatment and inflammation management.

## Data Availability

No datasets were generated or analysed during the current study.
